# Cinnamaldehyde Induces Release of Cholecystokinin and Glucagon-Like Peptide 1 by Interacting with Transient Receptor Potential Ankyrin 1 in a Porcine Ex-Vivo Intestinal Segment Model

**DOI:** 10.3390/ani11082262

**Published:** 2021-07-30

**Authors:** Elout Van Liefferinge, Maximiliano Müller, Noémie Van Noten, Jeroen Degroote, Shahram Niknafs, Eugeni Roura, Joris Michiels

**Affiliations:** 1 Laboratory for Animal Nutrition and Animal Product Quality (LANUPRO), Department of Animal Sciences and Aquatic Ecology, Ghent University, 9000 Ghent, Belgium; noemievannoten@gmail.com (N.V.N.); jerdgroo.degroote@ugent.be (J.D.); joris.michiels@ugent.be (J.M.); 2 Centre for Nutrition and Food Sciences, Queensland Alliance for Agriculture and Food Innovation, The University of Queensland, St. Lucia, QLD 4072, Australia; m.mullerbravo@uq.edu.au (M.M.); s.niknafs@uq.edu.au (S.N.); e.roura@uq.edu.au (E.R.)

**Keywords:** TRPA1, TRPV1, essential oils, ex-vivo model, cinnamaldehyde, capsaicin, gastric emptying

## Abstract

**Simple Summary:**

The gut is able to “sense” nutrients and release gut hormones to regulate digestive processes. Accordingly, various gastrointestinal cell types possess transient receptor potential channels, cation channels involved in somatosensation, thermoregulation and the sensing of pungent and spicy substances. Recent research shows that both channels are expressed in enteroendocrine cell types responsible for the release of gut peptide hormones such as Cholecystokinin (CCK) and Glucagon-like Peptide-1 (GLP-1). A large array of herbal compounds, used in pig nutrition mostly for their antibacterial and antioxidant properties, are able to activate these channels. Cinnamaldehyde, occurring in the bark of cinnamon trees, acts as an agonist of Transient Receptor Potential Ankyrin 1 (TRPA1)-channel. Capsaicin, the active component of chili peppers, acts as an agonist of Transient Receptor Potential Vanilloid 1 (TRPV1)-channel. This study explored the ability of both compounds to stimulate the release of CCK and GLP-1, and whether this response was mediated by TRP channels, using an ex-vivo intestinal segment model. Results showed mainly the potential of cinnamaldehyde to interact with TRPA1 and trigger CCK and GLP-1 release, while yet being dependent on the location in the small intestine.

**Abstract:**

Cinnamaldehyde and capsaicin have been reported to exert effects on the gastric function, mediated by the interaction with transient receptor potential ankyrin channel 1 (TRPA1) and transient receptor potential vanilloid channel 1 (TRPV1), respectively. This study examined whether these compounds could trigger the release of cholecystokinin (CCK) and/or glucagon-like peptide 1 (GLP-1) in the pig’s gut in a porcine ex-vivo intestinal segment model. Furthermore, it was verified whether this response was mediated by TRPA1 or TRPV1 by using the channel’s antagonist. These gut peptides play a key role in the “intestinal brake”, a feedback mechanism that influences the function of proximal parts of the gut. Structural analogues of cinnamaldehyde were screened as well, to explore structure-dependent activation. Results showed a significant effect of capsaicin on GLP-1 release in the proximal small intestine, TRPV1 independent. TRPA1 showed to be strongly activated by cinnamaldehyde, both in proximal and distal small intestine, evidenced by the release of CCK and GLP-1, respectively. Out of all structural derivates, cinnamaldehyde showed the highest affinity for TRPA1, which elucidates the importance of the α,β-unsaturated aldehyde moiety. In conclusion, cinnamaldehyde as a TRPA1 agonist, is a promising candidate to modulate gastric function, by activating intestinal brake mechanisms.

## 1. Introduction

Herbs and spices have been used since ancient times to enhance the taste and flavour of food and to stimulate digestive functions [1]. In addition, they are known for their antibacterial and antioxidant properties. For these benefits, they are used in small amounts in animal feed. These components are known to improve the animal’s overall health and performance, yet the application in the feed is mostly motivated by the antimicrobial and antioxidant properties they may have [2,3,4]. Interestingly, literature suggests improved digestibility of energy and nutrients with the supplementation of essential oils. For example, weaned pigs fed a diet supplemented with 0.01% of an essential oil product containing thymol and cinnamaldehyde significantly improved apparent total tract digestibility of dry matter and crude protein [5]. Ahmed et al. (2013) showed positive effects of a blend of oregano, anise, orange peel and chicory essential oils on protein digestibility in weaned piglets [6]. The same effect was shown in grower-finisher pigs fed a mixture of essential oils (0.01%) [7]. The improved apparent digestibility could be attributed to the enhanced secretion of bile acids and a stimulation of digestive enzyme activities. For example, curcumin, capsaicin, piperine, ginger, fenugreek and asafoetida enhanced activities of pancreatic lipase, pancreatic amylase, trypsin and chymotrypsin in rats [8]. Also, saline solutions containing capsaicin (50–1000 µg kg^−1^ BW) progressively increased acid output in the stomach of rats and could thus contribute to aid digestion by greater acidification which is associated with increased protein hydrolysis in the gastric fraction [9,10]. A plant extract mixture containing carvacrol, cinnamaldehyde and capsicum oleoresin, fed to weaned pigs, increased stomach content concomitant to a higher dry matter percentage, suggesting an increased gastric retention time [11]. Fledderus et al. (2007) showed that a 10% slower gastric emptying rate caused by the inclusion of 1% carboxymethylcellulose, resulted in an increased protein hydrolysis in the gastric fraction of the pig [12]. Modulating gastric emptying could be especially of interest around the weaning period, as brush-border enzyme activities and macronutrient digestion in the small intestine are reduced and more undigested carbohydrates and proteins are expected to reach the distal GIT in the period after weaning. It has been suggested that distal protein fermentation may selectively favor the proliferation of ETEC and Clostridia in the large intestine and predispose the occurrence of post-weaning diarrhea [13,14]. Nevertheless, specific evidence about the mode of action of phytogenic compounds to aid digestion is still scarce, particularly in the pig. The gastrointestinal tract (GIT) is able to efficiently absorb nutrients implying the existence of a luminal sensing mechanism of nutrients that has been referred to as the gut chemosensory system [15]. A possible chemosensory mechanism for phytogenic compounds could be the interaction with transient receptor potential (TRP) channels, cationic channels involved in a range of physiological processes such as pain, inflammation and thermoregulation. Several phytogenic compounds are known to activate these excitatory ion channels. As such, it has been shown that pungent natural compounds present in cinnamon oil, wintergreen oil, clove oil, mustard oil, and ginger all activate transient receptor potential ankyrin 1 (TRPA1) [16]. Being a member of the TRP family, TRPA1 is known to be involved in chemesthesis, the chemosensory pathway associated with pungent ingredients [17]. As such, Fothergill et al. (2016) showed that the increased transmucosal ion current in the mouse duodenum and colon, caused by allyl isothiocyanate (AITC), was reduced by the TRPA1 antagonist HC-030031 [18]. Transient receptor potential vanilloid 1 (TRPV1) was shown to be activated by capsaicin in a concentration-dependent manner, and the evoked response decreased by the addition of capsazepine, a TRPV1 antagonist [19]. Recent identification of TRPA1 expression in non-neuronal cells of the pig such as enteroendocrine cells, was interpreted as evidence of additional functions including the ability to modulate gastric function [20]. According to Nozawa et al. (2009), treatment of enterochromaffin cells with TRPA1 agonist AITC (300 µmol L^−1^) and cinnamaldehyde (300 µmol L^−1^) resulted in the release of serotonin [21]. Also, stimulation of STC-1 cells with AITC, increased intracellular calcium and significantly stimulated cholecystokinin (CCK) secretion [22]. In a MGN3-1 cell line, a ghrelin secreting cell model, the incubation with cinnamaldehyde (100 µmol L^−1^) upregulated the expression of TRPA1 and insulin receptor genes. Furthermore, stimulation with this compound resulted in a decrease in ghrelin secretion, which was partially recovered by the inhibition of TRPA1 with HC-030031 [23]. Emery et al. (2015) showed that TRPA1 activation caused an increase in glucagon-like peptide 1 (GLP-1) secretion from primary murine intestinal cultures and GLUTag cells [24]. Since compounds derived from herbs and spices are exposed to the mucosal lining of the GIT and previous research has shown that TRPA1 and TRPV1 are expressed on endocrine cell types in the pig’s stomach and small intestine (SI), the interaction between a phytogenic compound and TRPA1 or TRPV1 could enable the GIT to ‘sense’ spices and plant compounds and potentially to orchestrate GIT motility and the release of gut hormones involved in the regulation of digestive processes, energy and glucose homeostasis. The purpose of this study was to investigate whether cinnamaldehyde (a TRPA1 agonist) and capsaicin (a TRPV1 agonist) could induce a response to CCK and GLP-1 secretion, two key gut peptides in regulating gastric function, and whether this response was mediated by TRP channels. The release of these hormones results in decreased gastric emptying and activation of the intestinal brake mechanism. The ability to trigger this physiological feedback mechanism by nutrients at a specific location is poorly explored. Therefore, in the first experiment, the ability of both phytogenic compounds to release these gut peptide hormones in the porcine proximal SI was tested. Also, gene expression of TRPA1, TRPV1, GLP-1(encoded by the proglucagon gene) and CCK (encoded by preprocholecystokinin) was carried out. A second experiment was performed to validate whether cinnamaldehyde could trigger gut peptide secretion in the distal SI, in comparison to the proximal SI. A final experiment was set up to explore which structural features of cinnamaldehyde are important for the activation of TRPA1 in the proximal SI, resulting in the release of CCK. For this purpose, we used the porcine ex vivo intestinal segment model described by Ripken et al. (2014) and Voortman et al. (2012) which enables a more high-throughput screening of different candidates and thus the inclusion of more controls, to verify whether the response is TRP-channel specific [25,26].

## 2. Materials and Methods

### 2.1. Chemicals

#### 2.1.1. First Experiment

Both agonist candidates (cinnamaldehyde, natural, ≥95%, Food grade; capsaicin, ≥95%) and their blockers (HC-030031, ≥98%, HPLC-grade; capsazepine, ≥98%, HPLC-grade) were obtained from Sigma-Aldrich, Castle Hill, New South Wales, Australia. Dimethyl sulfoxide (DMSO) and phenylmethanesulfonylfluoride (PMSF) were purchased from Sigma-Aldrich (Castle Hill, New South Wales, Australia). Chemicals to make Krebs-Ringer buffer (KRB) (D-glucose (1.8 g L^−1^), magnesium chloride (0.0468 g L^−1^), potassium chloride (0.34 g L^−1^), sodium chloride (7.0 g L^−1^), sodium phosphate dibasic (0.1 g L^−1^), sodium phosphate monobasic (0.18 g L^−1^), HEPES (5.957 g L^−1^)) were purchased from Sigma-Aldrich (Castle Hill, New South Wales, Australia). The pH of the KRB buffer was adjusted to pH 7.4 before use.

#### 2.1.2. Second and Third Experiment

Agonist (cinnamaldehyde, natural, ≥95%, FG-grade) and the blocker (HC-030031, ≥98%, HPLC-grade) were obtained from Sigma-Aldrich, Overijse, Belgium. Derivates (2-methoxycinamaldehyde, >96%, FG-grade; 4-methoxycinnamaldehyde, >96%, FG-grade; hydrocinnamaldehyde, >90%, FCC-grade; cinnamic acid >99%) were obtained from Sigma-Aldrich (Overijse, Belgium). Dimethyl sulfoxide (DMSO) and phenylmethanesulfonylfluoride (PMSF) were also purchased from Sigma-Aldrich (Overijse, Belgium) as well as. Chemicals to make Krebs-Ringer buffer (KRB), as described in the previous section, were purchased from the same company. Cytotoxicity detection kit (PLUS) LDH (Roche Diagnostics, Indianapolis, IN, USA) was also purchased from Sigma-Aldrich.

### 2.2. First Experiment

#### 2.2.1. Animals and Sampling Procedures

Experimental procedures involving animals were approved by The University of Queensland (AEC approval Number CNFS/587/16). Six 25 days old (d0) piglets (Domestic Landrace × Large White, 3 males and 3 females) of 6.82 ± 0.85 kg were weaned from Sunpork farm (Westbrook, Queensland, Australia) and moved into a stable with environmental automatic control and plastic fully slatted floor pens at Herston Medical Research Centre (HMRC) (Herston Campus, The University of Queensland, Australia). Piglets were weighted, marked and assigned into 6 pens (1 pig/pen). Temperature was set between 27–28 °C. Piglets had ad libitum access to water and a commercial starter diet. On day 21 of the trial, piglets (13.99 ± 2.41 kg) were sacrificed by intravenous injection of sodium pentobarbital (200 mg kg^−1^). GIT-tissue segments from duodenum (1 cm distal from the pylorus), were collected within 10 min, stored in ice cold KRB/HEPES buffer bubbled with O_2_/CO_2_ (95%/5%), to prevent ischemia, and transported within 20 min to the lab to be used in a porcine intestinal segment model previously described by Voortman et al. (2012) [26] and Ripken et al. (2014) [25] with some minor changes. In short, once in the lab, tissues were immediately rinsed with cold KRB buffer, had their outer muscle layer stripped off and were cut open longitudinally. Equally sized round tissue samples of 12 mm in diameter (approximately 1.13 cm^2^) were punched out using a biopsy punch. Each circular tissue sample was then transferred into a 24 well-plate with the apical side upward, filled with 500 μL ice cold KRB/HEPES buffer at pH 7.4. The plates were brought to room temperature within 20 min, followed by a pre-incubation at 37 °C for 1 h in a humidified incubator, O_2_/CO_2_ (95%/5%). The KRB/HEPES buffer was then replaced by a pre-warmed KRB/HEPES buffer (37 °C, 500 μL with pH 7.4) without glucose but containing one of treatments (CIN: 200 μmol L^−1^ cinnamaldehyde, CAP: 20 μmol L^−1^ capsaicin, CIN-: 200 μmol L^−1^ cinnamaldehyde and 200 μmol L^−1^ HC-030031, CAP-: 20 μmol L^−1^ capsaicin and 20 μmol L^−1^ capsazepine) or a control (buffer, KRB) and incubated for an additional 1 h at 37 °C, O_2_/CO_2_ (95%/5%). All treatments were tested in each animal in triplicate, using a different 24-well plate per animal. Concentrations of test components were based on literature [18,27,28,29]. Due to their low solubility in water, test compounds were dissolved in KRB buffer containing less than 0.0125% DMSO. After incubation, the media from each well were collected into tubes and 100 μmol L^−1^ of PMSF was added to inactivate serine protease activity and avoid the cleavage of active GLP-1. In order to check tissue viability, media samples were collected to measure lactate dehydrogenase (LDH) activity (cytosolic enzyme released following cell abrasion). The collected media for hormone analysis were stored at −80 °C, whereas media samples for LDH activity were stored at 4 °C before analysis on the day itself. Tissue samples were stored in RNA-later in −80 °C for gene expression studies.

#### 2.2.2. Tissue Viability

Viability of tissues was monitored by measuring the intracellular LDH leakage into the medium to make sure that the release of hormones is not secondary to cell lysis or other nonspecific toxic effects. To determine the integrity of the intestinal tissue, enzyme activity was first determined as the total intracellular LDH activity, by homogenizing the tissue in ice cold KRB buffer using a tissue ruptor (Qiagen) for 5 min at 200 rpm (positive control). Next, leakage for all 4 treatments was determined by assessing LDH in supernatants and expressed as % of positive control. LDH activity was determined using a Roche LDH reagent kit PLUS (Roche Diagnostics, Indianapolis, IN, USA) according to the manufacturer’s instructions.

#### 2.2.3. Hormone Analysis

GLP-1 and CCK hormone levels were analysed using commercially available ELISA kits, according to the manufacturer’s instructions. Concentrations of CCK-58 were analysed using a Porcine Cholecystokinin ELISA kit from MyBioSource, Victoria, Australia (#MBS164396). Inter-assay coefficient of variation for the CCK kit was 7.5% and intra-assay coefficient of variation was 3.2%. GLP-1 release was measured using a GLP-1 (Total) ELISA kit from Millipore, Billerica, USA (EZGLPT1-36K). Inter-assay coefficient of variation for the GLP-1 kit was 7.8% and intra-assay coefficient of variation was 2.1%. When necessary, samples were diluted in assay buffer in order to obtain values within the detection range of this kit. Both hormone levels were measured on a BMG FLUOstar OPTIMA Microplate Reader.

#### 2.2.4. Gene Expression

Mucosal total RNA was isolated from the ex-vivo tissue samples kept in RNA later (KRB, CIN and CAP), using the Invitrogen PureLink^TM^ RNA Mini Kit (Invitrogen, Carlsbad, CA, USA). The concentration (ranging between 500 and 1200 ng mL^−1^) and purity (optical density 260A/280A ranging between 1.95 and 2.25) were analysed with the NanoDrop ND-1000 (NanoDrop Technologies, Thermo Scientific, Wilmington, DE, USA). The 18S and 28S bands were evaluated by loading on a 0.8% agarose gel, in order to check the RNA integrity. Sharp bands, including a background smear, were required to be visible. QuantiTect Reverse Transcription Kit (QIAGEN, Hilden, Germany) was used according to the manufacturer’s instructions for synthesizing cDNA. Briefly, 1 µg of RNA was mixed with 2 µL of gDNA Wipeout buffer and reached to 14 µL volume by adding RNase-free water. The mixture was incubated at 42 °C for 2 min to eliminate the genomic DNA in the samples. The absence of gDNA contamination was verified by performing a minus reverse transcription control using *YWHAZ* primers (Table 1). Then, 1 µg gDNA free RNA from each sample was converted to cDNA by using the Quantitect Reverse Transcription (RT) kit (Qiagen, Melbourne, Australia). The obtained cDNA was diluted 6 times with molecular grade water. A cDNA pool was prepared and specificity of all the primers (Table 1) were checked before real-time PCR. Finally, a verification of the reverse transcription reaction was performed through a control PCR using 1 µL of the diluted cDNA and the same primer as previously mentioned. Primers used for the target genes were designed using NCBI’s PrimerBLAST, based on the common sequence which will amplify all the transcript variants of the genes listed. The secondary structures in the target sequences were analysed using mfold [30]. Total volume of 10 µL (1 µL cDNA, 1 µL primers (2 µmol L^−1^ each), 3 µL water, and 5 µL SYBR green) was used for real-time PCR reactions. Samples were run in triplicate, and three reference genes *HPRT1*, *YWHAZ* and *GAPDH* were used to normalize the data. Positive controls for each gene using pooled cDNA with three technical replicates was considered in all the plates. Plates were run on Applied Biosystems RT-qPCR instrument (Applied Biosystems, Foster City, California, United States) with the following program: 50 °C for 2 min, 95 °C for 10 min, and 40 cycles of 95 °C for 15 s and 60 °C for 1 min. The melt curve stage was done under the following conditions: 95 °C for 15 s, 60 °C for 1 min with increment rate of 0.05 °C/s to reach 95 °C for 15 s. RT-qPCR data was analysed using the method according to Vandesompele et al. (2002) [31]. To do so, the PCR efficiency of each primer was calculated using delta Rn information which was calculated using the LinRegPCR software [32]. The Cq values were transformed into quantities, by using the delta-Cq formula; ${\left(1+\frac{efficiency}{100}\right)}^{∆CT\left(highest-sample\right)}$ with the highest expression level set to 1. The relative expression for each target gene was expressed as a ratio of the transformed Cq-value from the target gene to the geometric mean of the transformed Cq-values from the three stable expressed reference genes. The selection of reference genes was done by using three commonly used reference genes in pig stomach and small intestine: *HPRT1*, *YWHAZ* and *GADPH* (Table 1) [33].

### 2.3. Second Experiment

#### 2.3.1. Animals and Sampling Procedures

The second and third experiment were conducted in accordance with the ethical standards and recommendations for the accommodation and care of laboratory animals, covered by the European Directive 2010/63/EU on the protection of animals used for scientific purposes and the Belgian Royal Decree KB29.05.13 on the use of animals for experimental studies. No ethical approval was required for this trial according to local legislation as animals were kept under farm practices without interventions causing harm equivalent to, or higher than, that caused by the introduction of a needle in accordance with good veterinary practice, and because animals were killed solely for the use of their organs or tissues (2010/63/EU). Six 45-day-old (three weeks post weaning) piglets (Topigs Norsvin × German Pietrain) with a mean body weight (BW) of 12.21 ± 1.32 kg were selected from a commercial herd (Farm Van Kerschaver, Maldegem, Belgium) and housed in groups of two per pen with full slated floors in the experimental facility of the Department of Animal Sciences and Aquatic Ecology of Ghent University (Melle, Belgium). The stable had a conventional ventilation scheme, ambient temperature at 24 °C, and an 18 light/6 dark schedule. Piglets had ad libitum access to water and a commercial starter diet. Piglets were humanely sacrificed by inducing electronarcosis, followed by exsanguination. To investigate the effect of cinnamaldehyde on CCK and GLP-1 release in the distal SI, GIT-tissue segments from duodenum (1 cm distal from the pylorus, same as first experiment) and ileum (75% of total SI-length), were collected within 10 min. Tissue was stored in ice cold KRB/HEPES buffer bubbled with O_2_/CO_2_ (95%/5%), to prevent ischemia, and immediately transported to the lab to be used in a porcine intestinal segment model, as previously described. Only treatments in the second incubation step were changed: The KRB/HEPES buffer was then replaced by a pre-warmed KRB/HEPES buffer (37 °C, 500 μL with pH 7.4) without glucose but containing one of treatments (CIN: 200 μmol L^−1^ cinnamaldehyde, CIN-: 200 μmol L^−1^ cinnamaldehyde and 200 μmol L^−1^ HC-030031) or a control (buffer, KRB) and incubated for an additional 1 h at 37 °C, O_2_/CO_2_ (95%/5%). All treatments were tested in each animal in triplicate, using a different 24-well plate per animal.

#### 2.3.2. Hormone Analysis

GLP-1 and CCK hormone levels were analysed using commercially available ELISA kits, according to the manufacturer’s instructions. Concentrations of CCK-58 were analysed using a Porcine Cholecystokinin ELISA kit from MyBioSource, San Diego, CA, USA (#MBS264395). Inter-assay coefficient of variation for the CCK kit was 7.5% and intra-assay coefficient of variation was 3.2%. GLP-1 release was measured using a Glucagon-Like Peptide-1 (Total) ELISA kit from Millipore, Billerica, MA, USA (EZGLPT1-36K). Inter-assay coefficient of variation for the GLP-1 kit was 7.8% and intra-assay coefficient of variation was 2.1%. When necessary, samples were diluted in assay buffer in order to obtain values within the detection range of this kit. Both hormone levels were measured on a TECAN INFINITE M NANO Microplate Reader.

#### 2.3.3. Tissue Viability

Viability of tissues was monitored as previously described, using a Roche LDH reagent kit PLUS (Roche Diagnostics, Indianapolis, IN, USA) according to the manufacturer’s instructions.

### 2.4. Third Experiment

#### 2.4.1. Animals and Sampling Procedures

Six 38-day-old piglets (Topigs Norsvin × German Pietrain, two weeks post weaning) with a mean body weight (BW) of 9.05 ± 0.42 kg were selected from a herd (Farm Van Kerschaver, Maldegem, Belgium) and housed in a group of two per pen with full slated floors. The stable had a conventional ventilation scheme, ambient temperature at 24 °C, and an 18 light/6 dark schedule. Piglets had ad libitum access to water and a standard starter diet. Piglets were humanely sacrificed by inducing electronarcosis followed by exsanguination.

To explore which structural features of cinnamaldehyde are important for the activation of TRPA1 in the duodenum of piglets, the effect on CCK release of cinnamaldehyde next to four structural derivates was investigated in the duodenum of piglets. As such, 2-methoxy cinnamaldehyde (CIN2M), 4-methoxycinnamaldehyde (CIN4M) (both introducing an electron donating group on the aromatic ring of cinnamaldehyde), hydrocinnamaldehyde (HCIN) (missing the α,β-unsaturated bond) and cinnamic acid (CINA) (organic acid, lack of aldehyde function) were tested next to cinnamaldehyde and a control (KRB). GIT-tissue segments from duodenum (1 cm distal from the pylorus) were collected within 10 min and processed as previously described. Only treatments in the second incubation step were changed: The KRB/HEPES buffer was then replaced by a pre-warmed KRB/HEPES buffer (37 °C, 500 μL with pH 7.4) without glucose but containing one of the treatments (CIN, CIN2M, CIN4M, HCIN, CINA: 200 μmol L^−1^) or a control (buffer, KRB) and incubated for an additional 1 h at 37 °C, O_2_/CO_2_ (95%/5%). All treatments were tested in each animal in triplicate, using a different 24-well plate per animal.

#### 2.4.2. Hormone Analysis

CCK hormone levels were analysed as previously described in the second experiment.

### 2.5. Statistics

Normality of data and homogeneity of variance were tested using the Brown–Forsyth test in SAS Enterprise Guide 6 (SAS Institute, Cary, NC, USA). Regarding experiment 1 and experiment 3, data were analysed using a one-way ANOVA according to the GLM procedure of SAS with a linear model that included effects of treatment (fixed) and pig (random). Regarding experiment 2, statistical analysis was performed using two-way ANOVA with the GLM procedure of SAS with a 2-level full factorial design: treatment, location in GIT, and interaction, using pig as a random factor. Multiple comparisons were performed by a Tukey test. Data were expressed as means and *p* < 0.05 was considered significant.

## 3. Results

### 3.1. First Experiment

#### 3.1.1. Viability of Ex-Vivo Intestinal Tissue

LDH leakage for samples was on average 8.9 ± 0.2% for KRB, 9.0 ± 0.7% for CIN, 9.9 ± 0.2 for CAP, 9.0 ± 0.6 for CIN-, and 9.3 ± 0.2 for CAP-. Following the criteria stated by Voortman et al. (2012) [26] and Ripken et al. (2014) [25] LDH leakage should not exceed 10%. Taking this into account, all intestinal samples were within the limit of LDH leakage and treatment did not have a toxic effect.

#### 3.1.2. Release of Gastrointestinal Hormones upon Exposure to Cinnamaldehyde and Capsaicin

**Figure 1:**
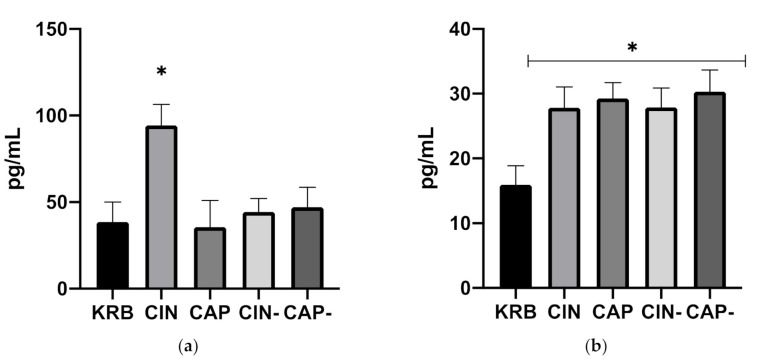
Effect of control KRB buffer (KRB), cinnamaldehyde (200 µmol L^−1^, CIN), capsaicin (20 µmol L^−1^, CAP), cinnamaldehyde (200 µmol L^−1^) + HC-030031(200 µmol L^−1^) (CIN-) and capsaicin (20 µmol L^−1^) + capsazepine (20 µmol L^−1^) (CAP-) on CCK (**a**) and GLP-1 (**b**) release. Significant differences compared to KRB are indicated with asterisks: * *p* < 0.05, number of replicates was *n* = 6, error bars express the SEM.

The basal secretion of CCK in duodenal tissue (KRB treatments) was higher than GLP-1 secretion (38.6 ± 15.2 pg mL^−1^ and 15.9 ± 11.1 pg mL^−1^, respectively). Figure 1a shows the effect of KRB, CIN, CAP, CIN- and CAP- on CCK secretion in the duodenum. Treatment had an effect (*p* = 0.008) on CCK release. Compared to the control treatment only CIN (94.4 ± 11.8 pg mL^−1^) stimulated CCK secretion (*p* < 0.05). CAP (35.6 ± 12.6 pg mL^−1^), CIN- (44.34 ± 11.8 pg mL^−1^) and CAP- (47.04 ± 10.6 pg mL^−1^) did not change CCK release as compared to the control, and no differences between these treatments were observed. Compared to CIN, CIN- (cinnamaldehyde incubated together with antagonist) CCK levels were almost 47% lower (*p* = 0.05), and at the same level as the basal CCK secretion. Figure 1b shows the effect of KRB, CIN, CAP, CIN-, and CAP- on GLP-1 secretion in the duodenum. Treatment had an effect (*p* = 0.007) on GLP-1 release. Compared to the control treatment (KRB), CIN (27.8 ± 12.4 pg mL^−1^), CAP (29.3 ± 9.5 pg mL^−1^), CIN- (27.9 ± 11.6 pg mL^−1^) and CAP- (30.3 ± 13.2 pg mL^−1^) significantly (*p* < 0.05) stimulated GLP-1 secretion resulting in concentrations that were almost two times higher as compared to control levels. No differences were observed between incubations with pure compounds (CIN, CAP) and with their respective antagonist (CIN-, CAP-).

#### 3.1.3. Gene Expression

**Figure 2:**
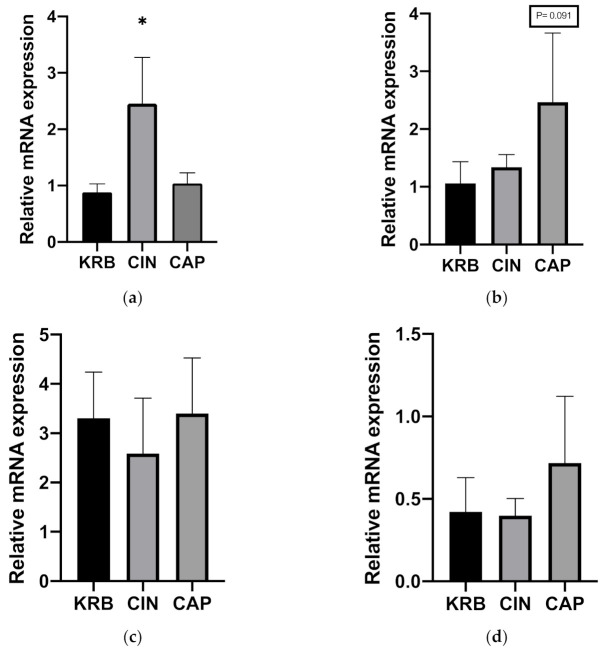
Effect of control KRB buffer (KRB), cinnamaldehyde (200 µmol L^−1^, CIN) and capsaicin (20 µmol L^−1^, CAP) on TRPA1 (**a**), TRPV1 (**b**), CCK (**c**) and GLP-1 (**d**) gene expression levels. Significant differences compared to KRB are indicated with asterisks: * *p* < 0.05, number of replicates was *n* = 6, error bars express the SEM.

The effect of CIN on the expression of several genes (TRPV1, TRPA1, CCK, and GLP-1) was determined. Addition of 200 µmol L^−1^ cinnamaldehyde (CIN) upregulated the expression of TRPA1 (*p* < 0.05) compared to KRB. The addition of 20 µmol L^−1^ capsaicin (CAP) did not alter TRPA1 mRNA expression (Figure 2a). In the case of TRPV1, there was a trend (*p* = 0.091) towards higher mRNA expression in tissue incubated with 20 µmol L^−1^ capsaicin (CAP) as compared to control (KRB) (Figure 2b). Incubation with CIN and CAP did not exhibit an effect on CCK and GLP-1 mRNA expression (Figure 2c,d).

### 3.2. Second Experiment

#### 3.2.1. Viability of Ex-Vivo Intestinal Tissue

LDH leakage for duodenal samples was on average 8.4 ± 0.3% for KRB, 7.2 ± 0.3% for CIN and 8.2 ± 0.7 CIN-. Ileum samples showed an LDH leakage of 8.7 ± 0.4% for KRB, 8.4 ± 0.3% for CIN and 8.5 ± 0.3 CIN-. All intestinal samples were within the limit of LDH leakage and treatment did not have a toxic effect.

#### 3.2.2. Release of Gastrointestinal Hormones upon Exposure to Cinnamaldehyde in Duodenum and Ileum

**Figure 3:**
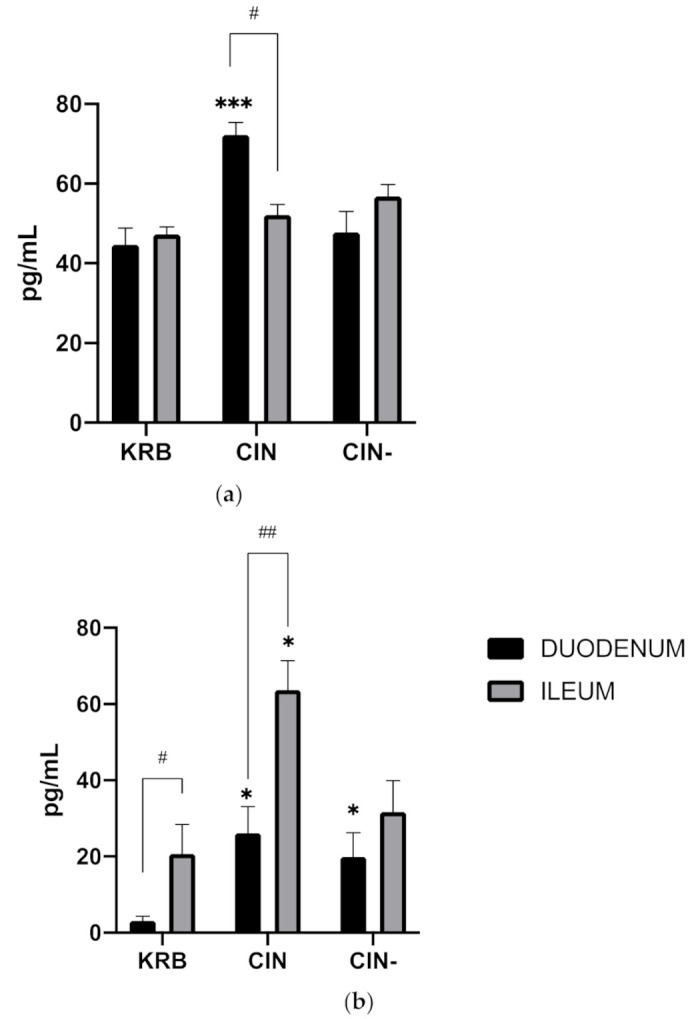
Effect of control KRB buffer (KRB), cinnamaldehyde (200 µmol L^−1^, CIN) and cinnamaldehyde (200 µmol L^−1^) + HC−030031(200 µmol L^−1^) (CIN-) on CCK (**a**) and GLP-1 (**b**) release. Significant differences compared to control (KRB) within the same intestinal site are indicated with asterisks: * *p* < 0.05, *** *p* < 0.001. Significant differences between intestinal sites within the same treatment are indicated with a hash: # *p* < 0.05, ## *p* < 0.01. *n* = 6, error bars express the SEM.

Figure 3a shows the effect of KRB, CIN and CIN- on CCK secretion in both the duodenum and ileum. Both treatment (*p* < 0.001) and the interaction (*p* < 0.001) have an effect. Compared to KRB, CIN potently stimulated CCK release (KRB, 44.7 ± 11.4 pg mL^−1^, CIN, 72.2 ± 11.1 pg mL^−1^, *p* < 0.01), in duodenal tissue (Figure 3). This response was completely blocked by incubating cinnamaldehyde together with the antagonist (CIN-, 47.7 ± 14.1 pg mL^−1^). No differences were observed between control (KRB) and treatments (CIN, CIN-) in the ileum regarding CCK release. Basal CCK secretion (KRB) in duodenum and ileum was around the same level (*p* = 0.995). Figure 3b shows the effect of KRB, CIN and CIN- on GLP-1 secretion in both the duodenum and ileum. Treatment (*p* < 0.001), and the interaction (*p* < 0.001) have an effect. Regarding GLP-1 in duodenal tissue (Figure 3), cinnamaldehyde (CIN, 26.2 ± 4.1 pg mL^−^1) stimulated release compared to control (KRB, 2.9 ± 2.4 pg mL^−1^, *p* < 0.05). This response was not blocked by incubating together with the antagonist (CIN-, 19.9 ± 6.4 pg mL^−1^). In ileal tissue, cinnamaldehyde evoked a higher response (CIN, 63.69 ± 16.6 pg L^−1^) compared to control (KRB, 23.4 ± 6.7 pg L^−1^, *p* < 0.001). This response was mostly blocked by incubating together with the antagonist (CIN-, 33.4 ± 6.7 pg L^−1^). Basal GLP−1 secretion (KRB) in ileum was higher compared to basal secretion in the duodenum (*p* < 0.05).

### 3.3. Third Experiment

#### Release of CCK upon Exposure to Cinnamaldehyde in Duodenum

**Figure 4:**
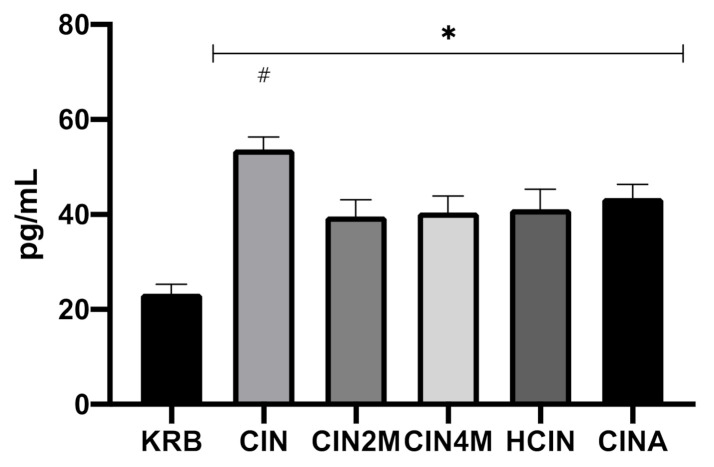
Effect of control KRB buffer (KRB), cinnamaldehyde (200 µmol L^−1^, CIN), 2-methoxycinnamaldehyde (200 µmol L^−1^, CIN2M), 4-methoxycinnamaldehyde (200 µmol L^−1^, CIN4M), hydrocinnamaldehyde (200 µmol L^−1^, HCIN) and cinnamic acid (200 µmol L^−1^, CINA) on CCK release in the duodenum. Significant differences compared to control (KRB) are indicated with asterisks: * *p* < 0.05. Significant differences compared to all treatments are indicated with a hash: # *p* < 0.05. Number of replicates was *n* = 6, error bars express the SEM.

Figure 4 shows the effect of KRB, CIN, CIN2M, CIN4M, HCIN, and CINA on CCK secretion in the duodenum. Treatment had an effect (*p* < 0.001) on CCK release. Compared to the control treatment (KRB, 23.4 ± 4.8 pg mL^−1^), all treatments (CIN: 53.87 ± 6.2 pg mL^−1^; CIN2M: 39.7 ± 8.4 pg mL^−1^; CIN4M: 40.4 ± 8.4 pg mL^−1^; HCIN 41.2 ± 10.2 pg mL^−1^; CINA: 43.5 ± 7.1 pg mL^−1^, *p* < 0.05), stimulated CCK release in duodenal tissue. Incubation with cinnamaldehyde (CIN) stimulated CCK release more potently compared to the derivates (*p* > 0.05); however, no differences were observed between incubation with derivates (CIN2M, CIN4M, HCIN, and CINA).

## 4. Discussion

Our study confirmed the hypothesis that cinnamaldehyde and capsaicin have an effect on GLP-1 and CCK release. As expected, basal CCK secretion in the proximal SI was higher compared to basal GLP-1 secretion. Voortman et al. (2012), observed a clear pattern of CCK, GLP-1, GLP-2 and PYY release along the porcine intestinal tract. CCK levels were predominantly released in the proximal small intestine from I cells. In contrast, tissue levels of GLP-1, known to be predominantly released from L cells in the ileum, are higher in the distal small intestine [26]. Cinnamaldehyde increased CCK release from the proximal small intestine. To our knowledge, this is the first report describing a direct effect of cinnamaldehyde on CCK release in the pig. These findings are in line with research performed by Purhonen et al. (2008), who showed a 6.7-fold higher secretion in STC-1 cells after stimulation with AITC, also known as a TRPA1 agonist [22]. In contrast, Bandell et al. (2004) showed that cinnamaldehyde had a higher affinity than AITC for TRPA1 [16]. However, tissue and cell disruption are expected to be higher in the ex-vivo intestinal model, which explains the smaller effect compared to control. Duodenal tissue incubated with both cinnamaldehyde and HC-030031, a selective TRPA1 antagonist, did not show a response in CCK release compared to the control indicating the mediating role of TRPA1.

Capsaicin did not trigger CCK release from duodenal tissue cultures. This pungent compound is known to stimulate TRPV1, which is expressed in the duodenum of the pig [20]. Since we have previously shown that TRPV1 was significantly more expressed in the distal small intestine of 28 days post weaning pigs [20], we suggest that targeting TRPV1 with capsaicin in the duodenum does not contribute in the release of CCK. However, previous results from our group showed a peak of expression of TRPV1 in the distal small intestine in post-weaning pigs, suggesting there might be an association with L cells, the most abundant cell type in this region [20]. L cells are responsible for GLP-1 release, which was significantly released after incubation with capsaicin. Nevertheless, incubation of duodenal tissue with capsaicin and the selective TRPV1 antagonist capsazepine, did not inhibit the response indicating that TRPV1 was not the mediating sensor. While TRPV1 gene expression showed a trend for a higher expression after treatment with capsaicin, this was not associated with GLP-1 release. Since the channel is expressed on enteroendocrine subsets, as shown in our previous findings [20], we speculate that capsaicin might be involved in the release of other gut hormones. In addition to capsaicin, cinnamaldehyde stimulated the release of GLP-1, but the response did not seem to be mediated by TRPA1. In contrast, Emery et al. (2015) showed an increase in GLP-1 secretion in GLUTag cells after treatment with carvacrol, which was abolished in cultures from TRPA1 knockout mice or pharmacological TRPA1 inhibition [24]. Instead of adding the agonist together with the antagonist, performing a pre-incubation with the antagonists would probably have been more effective. In this way the competitive antagonists capsazepine and HC-030031, would have been able to block TRPV1 and TRPA1 before adding the test compounds [34]. However, an additional pre-incubation step was discarded because it could have negatively affected tissue viability. The fact that the response triggered by cinnamaldehyde on CCK release was completely dampened by HC-030031 indicates that incubating the compounds together effectively blocks the channel.

Cinnamaldehyde upregulated TRPA1 mRNA expression, which highlights the molecule’s ability to activate this ion channel. TRPV1 gene expression shows a trend for a higher expression after treatment with capsaicin. In a study performed by Toschi et al. (2020), feeding 28-day-old piglets for 14 days with different doses of thymol (ranging from 25.5 mg kg^−1^ to 510 mg kg^−1^ feed) resulted in higher TRPV1 mRNA abundance in both duodenum and ileum [35]. Interestingly, thymol does not reach this part of the SI and gets quickly absorbed in the stomach and proximal SI [36].

Upon stimulation, peptide hormones, packed into secretory vesicles, are transported from the cytoplasm where they are synthesized to the secretion sites at the plasma membrane. Our results showed that cinnamaldehyde does not affect CCK mRNA expression level, yet stimulated CCK release mediated by TRPA1. Cinnamaldehyde-triggered activation of TRPA1 seems to play an important role in Ca^2+^ entry into the endocrine cells [18]. As a result of changes in cytoplasmic calcium concentrations, CCK is released from secretory granules by exocytosis. This process is characterized by cytoskeletal protein-mediated migration of the granules toward the cell surface, followed by their fusion with the plasma membrane and delivery of the hormones into the extracellular space [37]. As such, we presume that TRPA1 stimulation by cinnamaldehyde causes the docked peptidergic vesicles to immediately fuse to the plasma membrane and release their contents into the extracellular space. Based on the first experiment, cinnamaldehyde seems to be the most promising candidate to trigger GLP-1 and CCK release in the duodenum in pigs. The results obtained in the first experiment, confirmed the hypothesis that cinnamaldehyde triggers CCK release in the proximal SI in a TRPA1 dependent pathway. This exocrine function in the duodenum is part of the so called “duodenal break”, a negative feedback mechanism that influences the function of the proximal parts of the gastrointestinal tract. Activation of this feedback mechanism results in an enhanced and prolonged gastric distension, by inhibition of gastric emptying [38].

Cinnamaldehyde also showed to trigger GLP-1 release in the proximal SI. The primary site of expression of GLP-1 was found to be in the distal SI [26]. In addition, TRPA1 activation seems to play a role in the proximal feedback mechanism involved in the regulation of gastric emptying. Consequently, a second experiment was performed to confirm the previous findings. In this second experiment, cinnamaldehyde did not induce the release of CCK in the ileum. In this part of the porcine gut, low levels of CCK were observed [26]. Interestingly, the results obtained from the ileal tissue showed a significant release of GLP-1, which was mostly blocked by adding the TRPA1 blocker.

GLP-1 is an incretin hormone known to be released from intestinal L-cells postprandially. This gut derived peptide is able to normalize glycemia by promoting glucose dependent insulin secretion and inhibit glucagon secretion in the fasting state. However, the properties of GLP-1 as an enterogastrone, defined as a factor that slows gastric emptying and inhibits gastric acid secretion, have also been well described [39,40,41]. It has been suggested that the effect of GLP-1 slowing gastric emptying and delaying the delivery of nutrients into the small intestine, may outweigh its insulinotropic and glucagonostatic effects. The rate of gastric emptying influences postprandial glycaemic excursions, the fluctuations in blood sugar level, and vice versa [40]. In the distal part of the SI, the ileal brake is a feedback mechanism that results in inhibition of proximal gastrointestinal motility and secretion [42]. Results of our second experiment showing that TRPA1 activation by cinnamaldehyde results in the release of GLP-1, suggest the involvement of TRPA1 in this ileal feedback mechanism.

Cinnamaldehyde seems to affect both small intestinal brake mechanisms. Only few studies have been performed to compare the inhibitory effects of proximal (duodenal) and distal (ileal) exposure of nutrients on gastrointestinal motility. A study performed by Maljaars et al. (2007) showed that gastric emptying of a liquid meal was significantly delayed after ileal fat perfusion compared to an equicaloric duodenal infusion in man [38]. In dogs, gastric emptying and small intestinal motility was shown to be more strongly inhibited by an ileal perfusion (carbohydrate and fat) compared to a duodenal perfusion [43]. This suggests that access to the distal SI is necessary to obtain most potent inhibition of gastric emptying and motility. However, delivery of volatile compounds such as cinnamaldehyde to the distal small intestine is hard to tackle, due to their fast absorption in the stomach and proximal SI [36].

Results obtained from the third experiment highlighted which structural features are important to stimulate CCK release. TRPA1 contains ankyrin repeat motifs in the intracellular N-terminal moiety which possess cysteine and lysine residues, essential for activation by reactive agonists. TRPA1 could be activated by covalent binding of electrophiles to these cysteine residues, since the nucleophilic mercapto-group of cysteines is able to attack the α,β-unsaturated bond of cinnamaldehyde, via Michael addition [44].

To study the precise mechanism of covalent modification with unsaturated carbonyl-containing compounds, Sadofsky et al. (2011) examined a range of compounds which can undergo both conjugate and/or direct addition [45]. Results showed negligible TRPA1 activation with chemicals, only able to react with cysteines by conjugate addition such as cinnamic acid, weakly electrophilic at the carbonyl carbon. In contrast, compounds able to react via either conjugate or direct addition, such as cinnamaldehyde, showed a higher concentration-dependent activation. It was hypothesised in our study that less reactive derivates do not activate TRPA1 as potent as cinnamaldehyde, resulting in a decreased CCK response. Results obtained from our study showed that cinnamic acid does not stimulate CCK release as strongly as the aldehyde form. These results are in line with findings by Lieder et al. (2020), who showed that a range of naturally occurring compounds structurally related to cinnamaldehyde (cinnamyl alcohol, cinnamic acid, cinnamyl isobutyrate, and 2 methoxy-cinnamaldehyde) did not induce serotonin release to the same extent than cinnamaldehyde, in both Caco-2 and QGP-1 cell lines, by interacting with TRPA1 [46]. The aldehyde group seems more effective in activating TRPA1 compared to a carbonyl group, due to the electronic and steric profile of the compounds, which makes aldehydes more reactive toward nucleophilic substitutions. The introduction of an additional methoxy group (2-methoxycinnamaldehyde and 4-methoxycinnamaldehyde) also largely reduced the serotonin-releasing potential. A reason for this could lie in the change in the steric profile of the compound. A study performed by Nakajima et al. (2014) demonstrated that mono- and di- unsaturated aldehydes, but not saturated aldehydes, potently stimulate CCK secretion in the murine enteroendocrine cell line STC-1 [47]. This lower affinity was also seen in our study, since hydrocinnamaldehyde (missing the unsaturated bond) did not induce CCK release as strongly as cinnamaldehyde.

## 5. Conclusions

In conclusion, capsaicin stimulates GLP-1 release, but TRPV1 seemed not to be involved. It was demonstrated that cinnamaldehyde potentiated CCK release in the duodenum and GLP-1 release in the ileum by activation of TRPA1. Moreover, our findings elucidate that this compound, and not its structural derivatives missing the α,β-unsaturated aldehyde moiety, is a promising candidate to modulate gastric function in the pig, improving digestion.

## Figures and Tables

**Table 1:** Primers used for real-time PCR, annealing temperature (Ta), and amplicon length.

Gene Symbol	Accession Number	Nucleotide Sequence of Primers, 5′-3′	Ta (°C)	Product Length (bp)
*HPRT1*	DQ178126	Forward: CCGAGGATTTGGAAAAGGT Reverse: CTATTTCTGTTCAGTGCTTTGATGT	60	181
*YWHAZ*	DQ178130	Forward: ATGCAACCAACACATCCTATC Reverse: GCATTATTAGCGTGCTGTCTT	60	178
*GADPH*	NM_001206359.1	Forward: TGGTGAAGGTCGGAGTGAAC Reverse: GAAGGGGTCATTGATGGCGA	60	104
*TRPA1*	XM_021089237.1	Forward: GAATTTACTCATTGGTTTGGCAGTTGGTG Reverse: CGGTGATGGATTTCTGATCGACCTTG	58	155
*TRPV1*	XM_013981216.2	Forward: GGACAGCGAGTTCAAAGACC Reverse: CCGTTTTCCACCAGAAGTGT	63	240
*CCK*	XM_021068544.1	Forward: CAGGCTCGAAAAGCACCTTC Reverse: GCGGGGTCTTCTAGGAGGTA	60	157
*GLP-1*	XM_005671882.3	Forward: AGAACTCCGCCGCAGACA Reverse: TAAAGTCTCGGGTGGCAAGATT	60	83

## Data Availability

The data presented in this study are available on request from the corresponding author.
